# A Resume of the Present Status of the Cancer Problem

**Published:** 1916-01

**Authors:** Marion McH. Hull

**Affiliations:** Atlanta, Ga.


### A RESUME OF THE PRESENT STATUS OF THE CANCER PROBLEM.

By Marion McH. Hull, M. D., Atlanta, Ga.

In October it was my pleasure to spend a few days in Buffalo at the New York State Institute for the Study of Malignant Disease. This is the oldest institution in America for research work in the cancer problem, having been established through the efforts of the late Dr. Roswell Park. Its present director, Dr. Ivy R. Gaylord, has been in charge about sixteen years, and has gathered around him a group of workers who are so capable and conscientious that it is a real pleasure to be thrown with them. The impression which was left upon me after my association with them was most favorable, for they were very sure of what they knew because of a long line of experiments back of their statements, and do not make any extravagant claims.

Recently it was the pleasure of the Fulton County Medical Society to hear Dr. Gaylord speak of his work, and as he was here to see a patient of mine, I have been asked to summarize, for readers of this Journal, some of the conclusions which he presented. In doing so, I wish to disclaim in any sense being considered as Dr. Gaylord's spokesman. The statements which I make are the result of my impressions from his statements, and what I saw in their work.

I. They have definitely determined in the first place that cancer is not a disease, but a great group of diseases. For example, there are three distinct chicken sarcomas, each of which has its distinctive histological characteristic. One of these has the ordinary form; another in which there is a peculiar branching of the blood vessels; and a third, characterized by the formation of cartilage and bone. Each of these is caused by a filterable virus, which when injected into the tissues of the chicken forms its own peculiar type of disease. These viruses are different from each other. Each virus will produce its same type of disease, no matter what the tissue is that is injected. If the first is injected into connective tissues of the chicken, it will develop into the ordinary sarcoma; if the second is injected into the same connective tissue of the chicken, it will develop the branching type; if the third is injected into the same connective tissue of the chicken it will produce the third type, the osteochondrosarcoma. These are the only ones, the causative agents of which have been definitely determined; but these findings are sufficient to prove that cancer is not a disease, but a group of diseases. As further experimentation goes on, the causative agents of other diseases of the cancer group will be determined just as in this instance.

II. Cancer is probably the effort of the tissues to resist the invasion of the virus. When the virus of one of the chicken sarcomas is injected, the cells in the neighborhood of the injections begin to proliferate rapidly, and the growth is formed. Evidently the group is the effort of cells to protect the rest of the tissues from the invasion of the virus.

III. While it has never been possible to discover an organism which is responsible for the group of diseases which we call cancer, we know that there is an immunity which develops in the course of the disease. This immunity explains those cases of spontaneous recovery which are found at times in animals and, very rarely, in the human being. It has been found that in mice, a breast cancer will develop to a very large size sometimes, and then without anything having been done will begin to disappear, and the animal recover. The immunity to the disease has been developed, and is the only explanation which can be given for such a condition.

It has also been found that early in the disease, if a portion of the growth is removed by surgical procedure, the rest of it will disintegrate and disappear, and the animal recover.

It has also been the experience and observation that early in the disease if the growth be exposed to the X-ray or Radium recovery will take place. The only satisfactory explanation that can be offered for all of these facts is that immunity to the disease has developed.

IV. There is a specific immunity against the causative agents of cancer, which can be lowered or raised. The status of the immunity determines the appearance, the growth, or the disappearance of the disease. This immunity resides in the spleno-medullary apparatus. This fact is shown by the following experiments.

(a) It is impossible to implant cancer into a mouse which has recovered spontaneously.

(b) The injection of blood from a recovered mouse into another mouse renders this mouse so resistant that it is impossible to implant cancer in the second mouse.

(c) The injection of blood from a recovered mouse into the body of a mouse suffering with cancer, if not too far advanced, causes the disappearance of the cancer in the second mouse.

(d) In a mouse which has recovered from one type of cancer another type may be transplanted with success, although the original type cannot be transplanted. These facts prove the existence of a specific immunity.

V. This specific immunity can be lowered or raised by certain measures.

1. Exposing the spleen of a recovered mouse to the X-ray changes the immunity so that, whereas formerly it was resistant to the implanation of cancer, it now "takes" easily.

2. If you bleed a mouse formerly resistant, it becomes susceptible.

3. If you expose a recovered mouse to chloroform or other anaesthesia for any length of time, or successively during several days, the resistant mouse becomes susceptible.

4. Shock renders the resistant mouse susceptible.

Immunity can be raised.

1. By small doses of X-ray or radium.
2. By the injection of leucocytic extract.
3. By heat locally applied.
4. By improving the general metabolism.
5. By surgery judiciously used.

The status of knowledge with regard to the lowering of the resistance is without dispute. Work with regard to the means of raising the resistance is still in its experimental stage.

In the late stages of the diseases all known measures fail to raise the immunity. In other words, at this time there is no response of the spleno-medullary system to stimuli. Reasons for this will be explained in the next section.

VI. This immunity resides in the spleno-medullary apparatus. If you injure the spleen of an animal affected with cancer and doing well, the immunity fails and the growth begins to grow very rapidly. If you stimulate the spleen by the X-ray or in any other way, the immunity of the animal increases, and the tumor remains stationary or disappears.

In a long series of experiments in mice and in other lower animals, and also in human beings, these observations have been proven to be true; an increase in the lymphocytes is associated with an arrest or diminution of the tumor, whereas the decrease in the lymphocytes is associated with the growth of the tumor. In other words, when the spleno-medullary apparatus has been stimulated, the immunity is raised, and the tumor is arrested or diminishes. When the spleno-medullary apparatus is depressed, the immunity has been lowered, and the tumor grows, the animal becomes cachectic, and finally dies.

The lymphocytes themselves have no power over the growth, but constitute the index as to the activity of the spleno-medullary apparatus.

VII. The beneficial effect of the X-ray or radium, and to a certain extent of surgery, is due to the indirect effect on the system through the spleno-medullary apparatus. Surgery merely removes a focus of infection, and mobilizes the immunity, allowing the individual to utilize what immunity he has already, and limits the amount of work which the individual has to do in fighting the disease.

The X-ray and radium act, not as we formerly thought, by destroying the cancer cells, but by increasing the activity of the spleno-medullary apparatus. Within the last few weeks, Dr. Murphy, of the Rockefeller Institute, has shown this to be true which was claimed by Dr. Gaylord and his associates eleven years ago. Dr. Murphy took one hundred mice affected with cancer, removed the cancers, exposed them to the X-ray, and transplanted them in the axillae. Ninety-seven of these transplants took and grew. He then took one hundred other mice and removed the tumors, exposed the mice to the X-ray, but did not treat the tumors at all. He then transplanted these in the same way, and only three took. This proves conclusively that the X-ray did not have any effect whatever on the tumor itself, but on the individual through the spleno-medullary apparatus.

VIII. Practical conclusions from these very interesting facts which they have discovered might be briefly stated as follows:

1. Early in the disease the immunity is high. If operation is to be done, it should be done then.

2. Even though not all of the growth is removed (all should be removed that can be done without too serious damage to the surrounding structures) the rest of it will disappear, if the immunity is high.

3. Later on in the disease the status of the immunity should be determined before the second operation is performed. Physical harm can be done by the removal of the growth if the condition is such that the operation interferes with the immunity.

4. The X-ray should not be given in large doses. These depress the spleno-medullary apparatus and lower the immunity. Small doses over the spleen and thighs will give better results than large doses over the tumor.

5. There is some local effect, but the proper dose for local effect will probably not be the proper dose for the general effect.

6. What has been said in regard to the X-ray is also applicable to radium.

7. Every therapeutic measure should be controlled by the blood count.

8. All operations should be done under nitrous oxide and oxygen gas anaesthesia. The shock attendent upon local anaesthesia affects the immunity. Chloroform and ether anaesthesia lower the immunity.

9. Every effort should be made to avoid hemorrhage. The wound should be perfectly dry before it is closed up.

10. The cases for surgical interference should be carefully selected. Some cases are inoperable even though the growth is of such size that could be otherwise removed.

